# Classifying EEG for Brain-Computer Interface: Learning Optimal Filters for Dynamical System Features

**DOI:** 10.1155/2007/57180

**Published:** 2008-01-31

**Authors:** Le Song, Julien Epps

**Affiliations:** ^1^School of Information Technologies, The University of Sydney, N.S.W. 2006, Australia; ^2^National ICT Australia, Locked Bag 9013 Alexandria, N.S.W. 1435, Australia

## Abstract

Classification of multichannel EEG recordings during motor imagination has been exploited successfully for brain-computer interfaces (BCI). In this paper, we consider EEG signals as the outputs of a networked dynamical system (the cortex), and exploit synchronization features from the dynamical system for classification. Herein, we also propose a new framework for learning optimal filters automatically from the data, by employing a Fisher ratio criterion. Experimental evaluations comparing the proposed dynamical system features with the CSP and the AR features reveal their competitive performance during classification. Results also show the benefits of employing the spatial and the temporal filters optimized using the proposed learning approach.

## 1. INTRODUCTION

A brain-computer interface (BCI) is a communication system that relies on the brain rather than the body for control and feedback [1]. Ideally, it should run in a servo mode, allowing the subjects to initiate the communication anytime and anywhere without resorting to external stimuli or triggers. Such an interface not only offers a promising prosthetic device for those severely paralyzed, but also signifies a radically new technology for the general public. Current BCI research is still in its early stage and the emphasis is placed on the design of algorithms to decode a prespecified set of brain states. This involves three main aspects.

**Brain states**

Only brain states consciously controllable by the subjects are suitable for BCI. Besides, these states should generate distinct, repeatable, and measurable patterns whenever accessed. Among the most commonly used brain states are imaginations of body movements (motor imaginations). Motor imaginations can reliably change the neural activities over sensorimotor cortices. Depending on the part of the body imagined moving, these changes exhibit distinct spatial distributions [2]. Recognition of these patterns can then be translated into control signals, as is the case in this study.

**Recording devices**

Motor imaginations can be recorded by both electroencephalography (EEG) and magnetoencephalography (MEG). EEG remains the most popular way to record BCI signals and will be the focus of this study. It measures scalp electrical activities diffused from the cortex. Compared to MEG, it is portable and inexpensive. However, EEG can only measure blurred cortical activities due to the diffusion of the skull and the skin. Thus, EEG is normally used for studying cortical patches in the centimeter scale. Furthermore, EEG signals are contaminated by noise from various sources, such as muscle activities and power line interference. Spatial and temporal filters are commonly applied before any further analysis [3, 4].

**Decoding algorithms**

Prefiltered EEG signals still contain considerable noise, which poses a challenge for its decoding. Statistical machine learning (ML) techniques have been introduced into BCI to combat these variations. Techniques like artificial neural networks, support vector machine (SVM) [5], and Linear Discriminant Analysis [4] have been employed to learn patterns from training EEG signals and then classify new EEG signals. This strategy often results in increased decoding success and significant shortening of subject training time (from several months down to several days). The most prominent examples include the Berlin BCI [4], the MPI BCI [6], and the Graz BCI [7].

Apart from the classifiers, these ML-based BCIs also differ in the features they extract from EEG signals. The most successfully used features include autoregressive (AR) coefficients [6, 8] and common spatial patterns (CSP) [4, 7]. In this paper, we will employ a novel type of feature based explicitly on the neurophysiology of EEG signals instead. Basically, we consider EEG signals as the outputs of a networked dynamical system. The nodes of this system consist of cortical patches, while the links correspond to neural fibers. A large and complex system like this often generates interesting collective dynamics, such as synchronization in the activities of the nodes, and they result in the change of EEG patterns measured on the scalp. These features from the collective dynamics of the system can be employed for classification [9, 10]. This will be elaborated in Section 2.

To recover the cortical dynamics from the EEG signals, subject-specific spatial and temporal filtering is usually needed [4, 11]. Instead of manually tuning these filters, we propose a common framework in Section 3 to learn them from the data. Our basic idea is to optimize the filters so that the separability of the two classes is improved. Experimental results show that the learned filters not only reduce the classification errors of the dynamical system (DS) features, but also extract physically meaningful information from the EEG signals. Comparisons are also made between the DS features with the learned filters and the CSP and the AR features with manually tuned filters. These comparisons together with further comparisons to other filter learning methods, such as the CSSP [12] and CSSSP [4] methods, demonstrate the competitive performance of our method (Section 4). Finally, the conclusion is given in Section 6.

## 2. DYNAMICAL SYSTEM FEATURES

The cortex is a highly folded sheet of neurons (≈ 100 billion neurons) and they self-organize into clusters. These neuronal clusters not only tightly connect with their neighbors, but also communicate with distal clusters through neural fibers. Each cluster is often associated with certain aspect of information processing. The collaboration of these clusters achieves the normal functioning of the brain. In this section, we will first describe a simple mathematical model of the cortex, and then show how it leads to dynamical system features related to motor imaginations.

### 2.1. Mathematical model of the cortex

Typically, a neuronal cluster will generate electrical oscillations. It has been modeled as an oscillator with phase *θ* and output *s*. Its dynamics are governed by a simple phase model [13]:

(1) $$\begin{array}{c} s=f(\theta ),\\ \dot{\theta }=\omega +g(t), \end{array}$$

where *ω* is the intrinsic frequency of the oscillation and *f* is a function 2*π*-periodic in *θ*· *g*(*t*) is the input to the oscillator. g(*t*) will accelerate the oscillation if it assumes positive values, and slow it down if negative.

**Figure 1:**
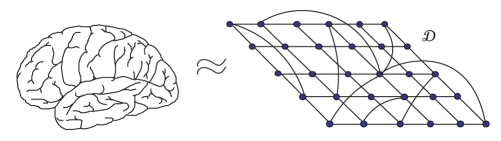
Networked dynamical system model of the cortex.

The whole cortex can then be modeled as a networked dynamical system *𝒟*, as shown in Figure 1. Each node in the system represents a neuronal cluster and each link a neural interaction. The input, *g*(*t*), to each neuronal cluster now consists of two parts: influence from other clusters and modulation by subcortical structures [2]. Suppose that the links of the network are represented as an adjacency matrix **G** (**G** *_ij_* = 1 if node *i* and *j* are connected; **G** *_ij_* = 0 otherwise). Then, the dynamics of a node *i* take a more specific form:

(2) $${\dot{\theta }}_{i}=\omega_{i}+\sum_{j}\varepsilon_{ij}G_{ij}(s_{j}-s_{i})+h_{i}(t),$$

where *s_i_* and *s_j_* denote the outputs from node *i* and *j*, respectively, ∑_*j*_ *ε*_*ij*_ **G**_*ij*_(*s* *_j_* − *s_i_*) represents the influence from other nodes, and *h_i_*(*t*) is the subcortical input. Note that there is an added parameter *ε_ij_* in (2), which controls the strength of the influence from node *j* to *i*.

### 2.2. Desynchronization of neuronal clusters

Two properties of the network of oscillators in (2) are of particular interest to BCI [13].

- Without the input *h*(*t*), all nodes will settle down into an oscillation of the same frequency *ω*_0_, if the network is connected and the influence *ε* is sufficiently strong (mutual synchronization).
- If the input *h_i_*(*t*) to node *i* is sufficiently strong and oscillates at a frequency *ω*_0_, node *i* will then be forced to oscillate in the same frequency *ω*_0_ (forced synchronization).

These two properties explain well the spatial distribution of the EEG signals during motor imaginations [2].

- If no imagination is carried out, the neuronal clusters in the idle sensorimotor cortex tend to synchronize with each other and oscillate in the frequency range of 8–26 Hz (EEG *α* and *β* rhythm). The spatial summation of this unison is a strong *α* (and/or *β*) rhythm in EEG signals.
- If the subject is actively engaged in motor imaginations, the associated neuronal clusters will be strongly modulated by the subcortical structures. The dynamics of these clusters will then stray away from their former synchronous state. This results in a decrease of *α* (and/or *β*) power in EEG signals.

This phenomenon is called event-related desynchronization (ERD) in the neuroscience literature. Depending on the part of the body imagined moving, neuronal clusters at different locations will be active. These clusters desynchronize with other clusters, and the spatial distribution of the desynchronization will be different as the imagination contents change. ERD suggests that the strength of the synchronization between neuronal clusters can be used as features for classification [9, 10].

### 2.3. Features for motor imaginations

An EEG electrode measures mostly the activities of the neuronal cluster directly underneath it (we will qualify this in Section 3). Suppose that the pairwise synchronization of the measured neuronal clusters can be computed from EEG signals and organized into a matrix **S** (**S** is symmetric with entry **S** *_ij_* for clusters *i* and _*j*_). Each entry in **S** is a dynamical system feature and the similarity between two EEG signals can then be quantified in terms of these features as follows:

(3) $$k(S,\overset{˜}{S})=Tr((S\circ A{)}^{⊤}(\overset{˜}{S}\circ A)),$$

where **A** is a weighting matrix, Tr(⋅) computes the trace of a matrix, and ∘ represents element-wise matrix product. Essentially, this measure transforms EEG trials into synchronization features and then computes their similarity based on these features. Since we will use a SVM classifier for our later experiments, *k*(⋅, ⋅) can be interpreted as a kernel between EEG trials.

Preliminary analysis of our motor imagination data set (this data set is further explained in Section 4) indicates that the synchronization in our data appears to be either inphase (*θ_i_* − *θ*_*j*_ = 0) or antiphase (*θ_i_* − *θ_j_* = *π*). These two types of synchronization can be well detected simply using the covariance. Therefore, classifying EEG signals using the DS features consists of three steps.

- Filter EEG signals. This is the step where filter learning techniques are applied. For our method, filters are learned for individual channels. Hence, EEG signals from different channels are filtered differently.
- Compute the entries of **S** and apply **A**. In this paper, **S** is simply the sample covariance matrix, and this is computed for each trial separately. Each entry in **S** is a DS feature, and the matrix **A** is mainly used for selecting the features. For instance, by setting 20 entries of **A** (in (3)) to 1 and all others to 0, then only 20 features are used for later classification.
- Compute the kernel *k*(⋅, ⋅) (in (3)) for pairs of trials, form the kernel matrix **K**, and pass it to SVM for classification. The entry in **K** corresponding to trial **S** and $\tilde{S}$ is simply *k*(**S**, $\tilde{S}$) (as in (3)).

## 3. LEARNING OPTIMAL FILTERS

Filtering EEG signals is important for later classifications. Due to the diffusion of the skull and skin, an EEG electrode actually measures a mixture of signals from several neuronal clusters. Spatial filters, such as a Laplacian filter, are usually applied to concentrate the signals to a single neuronal cluster. Furthermore, EEG signals are contaminated by various noises, such as electrical signals from muscle movements. Our interest lies in oscillation in the frequency range of 8–26 Hz (*α* and *β* rhythm). Bandpass filtering is usually needed to suppress other signals.

As previous BCI researchers have experienced [4], the optimal filters for each subject are very different, and it is quite inconvenient to manually choose these filters. Attempts have been made to learn these filters from the training EEG data. Pioneering works have been reported in [4, 12], where FIR (temporal) filters are learned for the CSP features to improve the separability of the two classes. Our work is inspired by their ideas, but our approach is different in two aspects. First, our approach is directed to the dynamical system features. Second, we have proposed a common framework for the learning of both the spatial and the temporal filters. In the following sections, the common framework is first described before it is specialized into the spatial and the temporal filter learning.

### 3.1. New framework

Our filter learning framework involves three steps: (i) quantify the quality of a feature using the Fisher ratio; (ii) express the Fisher ratio using the filter parameters; (iii) and then maximize the Fisher ratio with respect to the filter parameters. Given the data and the filter parameter **a**, our framework can be formulated mathematically as follows

(4) $$\underset{a}{\max}Q(a)=\frac{{\left(\mu_{+}(a)-\mu_{-}(a)\right)}^{2}}{\sigma_{+}^{2}(a)+\sigma_{-}^{2}(a)},$$

where *Q* is the fisher ratio, *μ* the mean value of a feature, and *σ* its standard deviation (the subscripts + and − restrict computation to positive and negative classes, resp.). Higher values of *Q* usually indicate better separation of the two classes. This learning framework can be applied to various problems. However, only local optimum can be guaranteed for the solution, since *Q* is in general not convex in terms of **a**. This is also the case in learning the filters for the DS features. To find an optimal solution efficiently, we will employ the subspace optimization technique.

The filter learning is performed on each pair of EEG electrodes separately. For a pair, two filters are learned, one for each electrode. Suppose that the parameters of the two filters are **a** and **b**, respectively. It turns out that for both the spatial and the temporal filtering, *Q* assumes a form biquadratic in **a** and **b**. For instance, if **b** is fixed, *Q* becomes the quotient between **a** ^⊤^ **V**(**b**)**a** and **a** ^⊤^ **W**(**b**)**a**, where **V**(**b**) and **W**(**b**) are matrices quadratic in **b**. The optimal **a** can then be obtained by solving the following constrained optimization problem:

(5) $$\begin{array}{l} \underset{a}{\max\;}a^{⊤}V(b)a,\;\;\text{s}\text{.t}\text{.}a^{⊤}W(b)a+\gamma b{}^{⊤}ba^{⊤}a=c. \end{array}$$

Note that the additional term *γ* **b** ^⊤^ **ba** ^⊤^ **a** does not originate from *Q*. It is a regularized product of the norms of **a** and **b**, and the strength of this regularization is controlled by *γ*.

Using the Lagrange multiplier method (let the multiplier be *λ*), the optimal **a** can be derived from the following generalized eigenvector problem:

(6) $$\tilde{V}(b)a=\lambda \tilde{W}(b)a,$$

where

(7) $$\begin{array}{c} \overset{˜}{V}(b)=V(b)+V{\left(b\right)}^{⊤},\\ \overset{˜}{W}(b)=W(b)+W{\left(b\right)}^{⊤}+2\gamma b^{⊤}bI. \end{array}$$

The optimal **a** is then the generalized eigenvector corresponding to the largest eigenvalue. Similarly, **b** can be optimized by fixing **a**. Local maxima can then be found by optimizing **a** and **b** alternately (Algorithm 1). In our experiments, the solution usually changes very little after two iterations, and henceforth only two iterations are used. To specialize this algorithm into the learning of the spatial and the temporal filters, we only need to derive the exact forms of **V**(**a**), **W**(**a**), **V**(**b**), and **W**(**b**) for these two cases, respectively.

### 3.2. Learning spatial filters

**Figure 2:**
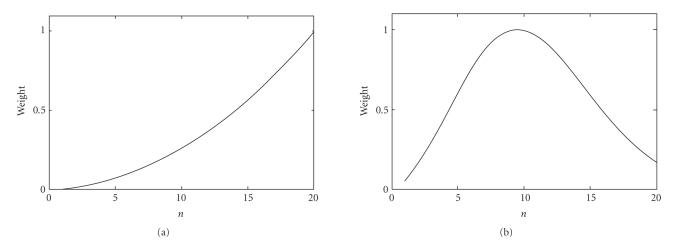
(a) Spherical spline Laplacian filter and (b) a bell-shaped filter.

Studies show that the spherical spline Laplacian filter is useful for the study of cortical dynamics [14]. This method models the shape of the head as a unit sphere and uses orthogonal bases on the sphere to spatially interpolate EEG signals [3]. The filtering is then achieved by computing the analytical Laplacian of the interpolation function. This filters only high-passes EEG signals, and is unable to emphasize interesting signals in the middle frequency range [11] (also see Figure 2). This section will start with a reformulation of the spherical spline Laplacian filter, which leads to a class of spatial filters. The exact forms of **V**(**a**), **W**(**a**), **V**(**b**), and **W**(**b**) are then derived.

For square integrable functions on a sphere, the Legendre polynomials *p_n_*(⋅) evaluated at cos*θ* constitute a set of orthogonal bases. The parameter *n* is the degree of the polynomial and it controls the spatial frequency of a basis. A *p_n_* with larger *n* will generally represent higher spatial frequency. *θ* is the latitudinal (zonal) angle. In this study, a maximum of *n* = 20 is used for the interpolation of EEG signals (due to the low spatial variation of EEG signals).

Suppose that a position on the unit sphere is **e**, and the position of the *i*th EEG electrode is **e**_*i*_. Let cos(**e**, **e**_*i*_) denote the cosine of the angle between **e** and **e** *_i_*, we can construct a matrix **P**(**e**) with entries:

(8) $${\left(P(e)\right)}_{in}=\frac{1}{4\pi }\frac{2n+1}{{\left(n(n+1)\right)}^{4}}p_{n}(\cos(e,e_{i})),$$

where *i* ranges through the index of the electrodes, and *n* = 1 ⋯ 20. Then, EEG signals at position **e** can be interpolated as follows:

(9) $$u(e)=c_{0}+C^{⊤}P(e)1,$$

where **u**(**e**) is a vector with each dimension corresponding to a time point. **1** is a vector of all ones. **c**_0_ (a vector of the same size as **u**(**e**)) and **C** ^⊤^ (a matrix with 20 columns and the same number of rows as **u**(**e**)) are the interpolation coefficients estimated from actual EEG signals. The solution of these coefficients can be found using two constraints [3]: (i) the interpolated function has to pass the actual EEG measurements; (ii) **C** ^⊤^ ⋅ **1** = **0**. Our formulation in (9) is equivalent to (1) in Perrin's original formulation [3]. The difference is that (9) describes the interpolation for each time point of a time series rather than that of a single time point. Our reformulation simply stacks separate interpolation for each time point into a matrix notation. This provides us with insight to how spatial filtering is performed.

Spatial filtering of EEG signals can then be achieved by simply removing the DC component **c**_0_ and reweighting other frequency components (the bases). Suppose that the filter (weighting) is **a**. Thus, spatial filtering can be computed as follows:

(10) $$\overset{˜}{u}(e_{i})=C^{⊤}P(e_{i})(1\circ a)=C^{⊤}P(e_{i})a.$$

The spherical spline Laplacian filter can be obtained by simply setting entries of **a** to −*n*(*n* + 1) (equivalent to [3], equation (5)). With formula (10), other types of filtering can also be implemented by varying **a**. For example, a bell-shaped bandpass filter can be obtained by setting the entries of **a** to exp(−*κn*(*n* + 1))*n*(*n* + 1) (*κ* is a parameter controlling the width and the peak). These two filters are illustrated in Figure 2. Note that the weights in the figures are normalized into the range between 0 and 1.

Suppose that filter **a** and **b** are applied to electrode **e**_*i*_ and **e**_*j*_, respectively, the covariance between the two filtered EEG signals can then be computed as

(11) $${\text{cov}}^{ij}=\frac{1}{l}\overset{˜}{u}{\left(e_{i}\right)}^{⊤}\overset{˜}{u}(e_{j})=\frac{1}{l}a^{⊤}P^{⊤}(e_{i})CC^{⊤}P(e_{j})b,$$

where *l* is the number of time points. Further, denote ${\overset{˜}{C}}^{ij}=P^{⊤}(e_{\text{i}})CC^{⊤}P(e_{j})$. (Since the following derivations are the same for each pair of electrodes, the superscripts *i j* are dropped henceforth for convenience.) Then, *μ*+ in (4) can be computed as follows:

(12) $$\mu_{+}=\frac{1}{m}\underset{k\in +}{\sum }{\text{cov}}_{k}=a^{⊤}(\frac{1}{ml}\underset{k\in +}{\sum }{\overset{˜}{C}}_{k})b=a^{⊤}D_{+}b,$$

where *k* *∈* + means that the index ranges through all *m* trials in the positive class. (Suppose that the negative class also has *m* trials). The variance *σ*+ can be computed as follows:

(13) $${\left(\sigma_{+}\right)}^{2}=\frac{1}{m}\underset{k\in +}{\sum }{\left({\text{cov}}_{k}-\mu_{+}\right)}^{2}=a^{⊤}E_{+}(b)a,$$

where

(14) $$E_{+}(b)=\frac{1}{m^{2}l^{2}}{\left((\sum_{k\in +}{\tilde{C}}_{k})b\right)}^{2}-\frac{1}{ml^{2}}\sum_{k\in +}{\left({\tilde{C}}_{k}b\right)}^{2}.$$

Similarly, *μ*_−_ = **a** ^⊤^ **D**_−_ **b** and (*σ*_−_)^2^ = **a** ^⊤^ **E**_−_(**b**)**a**. **V**(**b**) and **W**(**b**) can then be derived as follows:

(15) $$\begin{array}{c} {\left(\mu_{+}-\mu_{-}\right)}^{2}=a^{⊤}{\left(D_{+}b-D_{-}b\right)}^{2}a=a^{⊤}V(b)a,\\ {\left(\sigma_{+}\right)}^{2}+{\left(\sigma_{-}\right)}^{2}=a^{⊤}(e_{+}(b)+e_{-}(b))a=a^{⊤}W(b)a. \end{array}$$

Since **a** and **b** are symmetric, **V**(**a**) and **W**(**a**) can be derived analogously by exchanging the positions of **a** and **b** and transposing ${\tilde{C}}_{k}$ in (12)–(15). Substituting **V**(**a**), W(**a**), **V**(**b**), and **W**(**b**) into Algorithm 1 will then produce the optimal filters.

### 3.3. Learning temporal filters

Unlike [4] that formulated the learning of the temporal filters in the time domain (FIR filter), our formulation works directly in the frequency domain. The basic idea of our approach is to place weighting directly on the complex coefficients of the discrete Fourier transformation (DFT).

Weighting the frequency components of an EEG signal **u**(**e** *_i_*) will transform it to

(16) $$\tilde{u}(e_{i})={ℱ}^{-1}(ℱ(u(e_{i}))\circ a),$$

where **a** is the filter (weighting), and *ℱ* represents the forward DFT (*ℱ* ^−1^, the inverse DFT). Suppose that filters **a** and **b** are applied to EEG electrodes **e** *_i_* and **e** *_i_* , respectively. The covariance of the filtered signals can then be computed as follows:

(17) $$\begin{array}{ll} \text{cov} & =\frac{1}{l}\overset{˜}{u}{\left(e_{i}\right)}^{⊤}\overset{˜}{u}(e_{j})\\ & =\frac{1}{l}{\left({ℱ}^{-1}(ℱ(u(e_{i}))\circ a)\right)}^{⊤}({ℱ}^{-1}(ℱ(u(e_{i}))\circ b)). \end{array}$$

(Note that the superscripts are dropped for convenience.) Computation (17) is inefficient, since two forward and inverse DFTs are needed. The computation, however, can be reduced using the correlation theorem. This theorem states that the covariance between two signals **u**(**e**_*i*_) and **u**(**e**_*j*_) is equal to (*ℱ*(**u**(**e**_*i*_)))∗*ℱ*(**u**(**e** *_j_*)) (∗ denotes conjugate transpose). Thus, (17) can be simplified to:

(18) $$\text{cov}=\frac{1}{l}a^{⊤}\text{Diag}((ℱ(u(e_{\text{i}}{)))}^{*}\circ ℱ(u(e_{j})))b,$$

where Diag(⋅) transforms its vector argument into a diagonal matrix. Formula (18) requires only two DFT computations, and hence it is more efficient.

The derivations for **V**(**a**), **W**(**a**), **V**(**b**), and **W**(**b**) become straightforward if we compare (18) with (11). By setting ${\tilde{\text{C}}}^{ij}=\text{Diag}\left({\left(ℱ\left(u\left(e\right)\right)\right)}^{*}\circ ℱ\left(u\left(e_{j}\right)\right)\right)$, they can be obtained from (12)–(15). Substituting these matrices into Algorithm 1 produces the optimal filters.

## 4. RESULTS AND COMPARISON

Averaged dynamical system features (DS) and common spatial patterns (CSP) for five subjects (“aa,” “al,” “av,” “aw,” and “ay”) over three-time windows (0–0.5 second, 0.5–2.5 seconds, and 3.5–4.0 seconds) during the motor imagination. The top 20 most discriminative DS features are shown as edges connecting the corresponding electrodes (dots). The most discriminative CSPs for right-hand imagination are plotted as color maps.

The dynamical system (DS) features and the filter learning approach are evaluated using data set IVa from the Berlin BCI group [8]. This data set contains EEG signals (118 channels, sampled at 100 Hz) for five healthy subjects (labeled “aa,” “al,” “av,” “aw” and “ay,” resp.). During the recordings, they were prompted by visual cues to imagine for 3.5 seconds either right-hand (the positive class) or right-foot movements (the negative class). Our classification analysis will focus on the data between 0.5 seconds and 2.5 seconds (i.e., 200 time points for each channel), since in an online BCI setting a sliding window seldom exceeds 2 seconds [4]. For convenience, the period between 0 s and 1s of a trial is called imagination preparation stage, and the period between 3.5 seconds and 4.0 seconds is called postimagination stage. Each type of imagination was carried out 140 times. Thus, there are 280 labeled trials in total for each subject (note: each trial is a multivariate time series of 118 dimensions). The task is to classify the type of the imagination for each trial in an offline fashion.

Two sets of experiments were conducted in our evaluations. They are designed to reveal two major aspects: (i) using DS features for classification, and (ii) learning spatial and temporal filters. We will first describe procedures common to these experiments. All our classifications are carried out using SVM and the errors are obtained from 10 × 10 fold cross-validation. An identical temporal prefiltering (bandpass between 8–40 Hz) is applied to all subjects. In the case of the DS features, an identical spatial prefiltering (a bell-shaped bandpass filter exp(−*κn*(*n* + 1))*n*(*n* + 1) with *κ* = 0.01) is also applied for all subjects. Furthermore, only the top 20 DS features (in terms of their Fisher ratios) are used for classification.

### 4.1. Dynamical system features

#### 4.1.1. Where are the discriminative dynamical system features?

The dynamical system (DS) features during motor imagination (0.5–2.5 s) are scored by Fisher ratio for each fold of the cross-validation, and these scores are further averaged over the folds. The top 20 most discriminative DS features are plotted in the second column of Table 1. For comparison, typical common spatial patterns (CSPs) for the right-hand imagination (corresponding to the smallest generalized eigenvalues) are also shown beside the DS features.

For four of the five subjects (“aa,” “al,” “av,” and “ay”), the DS features share clear pattern across the subjects —they tightly concentrate on the area in charge of right-hand imagination (left motor cortex, hand region in the Homunculus). This phenomenon can be well explained by the theory of event-related desynchronization (ERD): as the hand region in the left motor cortex is actively engaged in imagination, its neuronal activities deviate from those of the neighboring cortices; and such localized spatial discordance results in the tight cluster of the DS features.

Furthermore, the typical common spatial patterns (CSP) also show nice agreement with the DS features. The areas of the ERD correspond to the peaks in the CSPs.

Beside the similarity also revealed in the figures is the difference of the DS features across subjects. Especially for subject “aw,” half of the DS features locate in the contralateral hand region. A plausible explanation is that the subject may have imagined movements of both hands.

#### 4.1.2. How do dynamical system features evolve over time?

The top 20 DS features in the imagination preparation stage (0–0.5 s) and the postimagination stage (3.5–4.0 s) are scored similarly and plotted, respectively, in the first and the third column of Table 1. These figures provide us an idea of the evolution of the DS features over time.

During the preparation stage, the DS features scatter around the whole scalp. They mostly connect distal regions of the brain; other than that, no clear pattern is shared across subjects. In fact, these DS features provide classifications only slightly better than random (the errors are not reported). This implies that the DS features within this period do not contain useful information for classification.

During the imagination, tight clusters of DS features are formed and they lead to good classification. Then, as the subjects are signaled to stop their imaginations (3.5–4.0 s), the clusters start to diffuse into wider areas of the brain. Such trend is most clearly revealed in subject “av,” where parts of the DS features are replaced by long range connections across hemispheres of the brain.

The formation and the dissolution of clusters over the course of an imagination present a unique characteristic for the DS features. Potentially, such pattern can be exploited for online detection of motor imagination.

#### 4.1.3. Dynamical system features are competitive

The DS features obtained with learned filters were compared to the CSP and the AR features obtained with manually chosen parameters. The parameters for the CSP features (filtering frequency, selected channels, and the number of projection subspaces) and the AR features (filtering frequency, selected channels, and the order of the AR model) were tuned according to the winning entry of BCI competition III [15]. The results are shown in Table 2.

Overall, the CSP features perform the best, the DS features follow, and the AR features produce lower accuracy. Furthermore, the DS features often obtain the best (highlighted in bold) or the second best place (highlighted in italic). Especially for subject “av,” the DS features outperform the CSP features by 6%. It is important to note that the parameters for the CSP and AR features have been tuned manually and intensively, while the results for the DS features are obtained with exactly the same starting parameters. This shows the usefulness of the DS features and our filter learning approach.

#### 4.1.4. Dynamical system features extract complementary information

The CSP, AR, and DS features are computed differently from the EEG signals. An interesting question is whether they complement each other during classification. To investigate this, we combine more than two types of features (CSP + AR, CSP + DS, AR + DS, and ALL three) using the META scheme described by [8]. The classifications of the combined features are presented in Table 3. The combination with the smallest error for each subject is highlighted in bold and the second place in italic. Furthermore, we surround an error with a box, if it is the smallest ever (in Tables 2 and 3) for a subject.

The DS features indeed complement the CSP and the AR features, as is evidenced by the further reduction of errors in subject, “aa,” “av,” and “aw.” The reduction, however, is not large (the largest being around 1% for subject “aa”). Furthermore, the combination of all three types of features does not necessarily further reduce the errors. This happens when the best features have already extracted almost all information about the separability of the two classes. Additional features may only provide redundant or even conflicting information for the classification. This is very likely in our case since we have optimized each type of features intensively. Finally, our results suggest that the combination of the CSP and the DS features performs the best, and the DS features complement the CSP features better than the AR features.

### 4.2. Learned filters

#### 4.2.1. Learned filters improve classification

For each pair of EEG electrodes (equivalent to a DS feature), the optimal spatial and temporal filters were learned sequentially. In Table 4, we present the classification errors using the following: (i) the DS features without the spatial and the temporal filter optimization (DS column); (ii) the DS features only with the spatial filter optimization (DS + S column); (iii) the DS features only with the temporal filter optimization (DS + T column); (iv) the DS features with both the spatial and the temporal filter optimization (DS + S + T column). Note that for all four comparisons prefilters have already been applied in both temporal and spatial domains.

The results demonstrate that both the learned spatial and temporal filters improve the classification (DS + S and DS + T columns). Although there is no absolute winner in the two types of filters, when applied separately, the temporal filters outperform the spatial filters in general (the winning filter for each subject is highlighted in bold). Especially for subjects “aa” and “aw,” the temporal filters reduce about 5% more errors than the spatial filters.

The combined application of the learned filters almost always further reduces the errors (only subject “av” slightly violates this rule). The maximum reduction is around 7% (for subject, “aa” and “aw”). The errors obtained (DS + S + T column) are now lower than 10% for 4 of the 5 subjects (except “av”). It seems that the learned filters help less for some subjects (“al” and “ay”). The reason can be that the prefiltering is already near the optimal solution.

The classification for subject “av” has the largest error. Our preliminary studies indicate that the most responsive frequency range of this subject shifts above 26 Hz (contrary to the usual 8–26 Hz). While most energy in the EEG signals concentrates below 26 Hz, this makes it difficult to extract good features for the subject.

#### 4.2.2. Learned filters extract meaningful information

Several details related to Section 4.2.1 are clarified here. The spatial and the temporal filters can be interpreted as weighting in the corresponding frequency domain. We have further restricted them to be polynomial models in our experiments. The results in Table 4 are obtained with polynomial functions of degree 6 (for both the spatial and the temporal filter learnings). The regularization parameters *γ* for the spatial and the temporal filters are 10^−7^ and 10^−13^, respectively. For the case of the temporal filter, a bell-shaped prefilter is also applied (− exp(− *κn*(*n* + 1))*n*(*n* + 1) with *κ* = 0.001 for all subjects). Note that the filters are always learned in pairs, that is, one for each channel in a pair. We will illustrate the learned filters in two ways.

**Figure 3:**
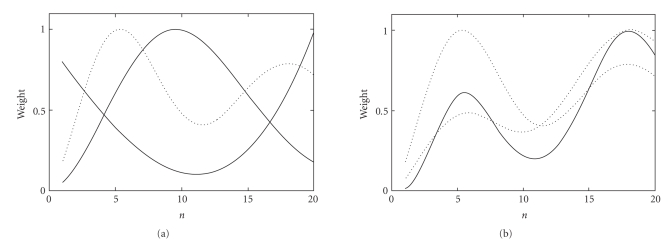
Illustration of spatial filters: (a) the prefilter (thin line, bell-shaped), a learned filter (thin line, bow-shaped), and their multiplication (dotted line); (b) learned filters from a pair of channels (dotted lines) and their multiplication (thick line).

**Figure 4:**
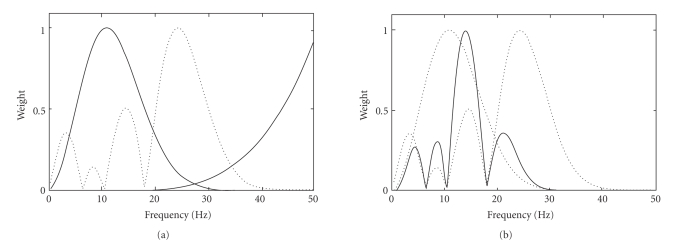
Illustration of temporal filters: (a) the pre-filter (thin line, bell-shaped), a learned filter (thin line, wedge-shaped), and their multiplication (dotted line); (b) learned filters from a pair of channels (dotted lines) and their multiplication (thick line).

The first way is the joint effect of the bell-shaped prefilter and a learned filter from a single channel. Since the learned filter is always applied after the prefiltering, we will show the shape of the prefilter, the learned filter, and their multiplication in one picture (Figures 3(a) and 4(a)).

The second way is the joint effect of the overall filtering from two channels. Since a DS feature is bilinear in the filters applied to the two channels, our optimization in Algorithm 1 only has exact control over their multiplicative effect. Therefore, we will illustrate the filtering effects for two channels and their multiplication in one picture (Figures 3(b) and 4(b)).

Figure 3(a) shows a learned spatial filter (thin line, bow-shaped) and the prefilter (thin line, bell-shaped) for one channel. Although both filters are simple, their multiplicative effect creates a double-peak characteristics (dotted line). This is equivalent to emphasizing the frequency contributions under these two peaks. The overall effect of the learned filters from two channels (dotted lines in Figure 3(b)) is also double peaked (thick line in Figure 3(b)). We believe that these peaks are somehow related to the electrode spacing on the scalp. It is likely that the learned filters weight the information from the actual electrodes more heavily than that from the interpolated positions.

For the temporal filters, we will interpret the learned filters in terms of their effects on the power spectrum. Hence, only the absolute values of the weighting are displayed. The final filter for an example channel (dotted line in Figure 4(a); it is the multiplication of a prefilter and a learned filter, both in thin lines) does not appear to emphasize the motor imagination signals (i.e., ERD in 8–26 Hz). The meaning, however, becomes clearer when we examine the filters from two channels together. In Figure 4(b), the filters from two channels are shown in dotted lines and their multiplication in thick line. The multiplication creates the strongest peak within 10–18 Hz, and a second strongest peak within 18–28 Hz. This corresponds well to the most responsive frequency range of the motor imaginations.

Note that one can *not* simply replace individual filters, **a** and **b**, from a pair of electrodes by the square root of their multiplication. This is because the two filters **a** and **b** always appear in the form of **ba** ^⊤^ in the objective and the constraint of (5). For instance, one can show that according to (15)

(19) $$aV\left(b\right)a=\text{T}\text{r}\left({\left(\left(D_{+}-D_{-}\right)ba^{⊤}\right)}^{2}\right).$$

Therefore, only when two pairs of filters, **a** and **b** versus **a**′ and **b**′, produce the same outer product (i.e., **ba** ^⊤^ = **b**′ **a**′^⊤^), they can be equated with each other. In Figures 3(b) and 4(b), we only showed the diagonal of **ba** ^⊤^ to produce a concise summary of their joint filtering effect. One should keep in mind that the learned filters have further effect beyond what is visualized here.

#### 4.2.3. Learned filters are competitive

The DS features obtained with the learned filters were compared to the CSP features produced by the CSSP [12] and the CSSSP [4] methods. These two methods are also designed to remove the manual filter tuning, and they have incorporated the filter learning into the original CSP method. The comparisons are presented in Table 5.

It can be seen that the three methods are quite competitive. Each method has its best performance in certain subjects. Notably, our method does the best in subject “av,” outperforming the other two methods by about 10%. As mentioned earlier, the most responsive frequency range of “av” shifts above the normal *α* and *β* bands (8–26 Hz). This seems to suggest that for such BCI “abnormal,” the DS features may be a better choice for the classification task.

## 5. DISCUSSION

### 5.1. Relation to other filter learning methods

**Figure 5:**
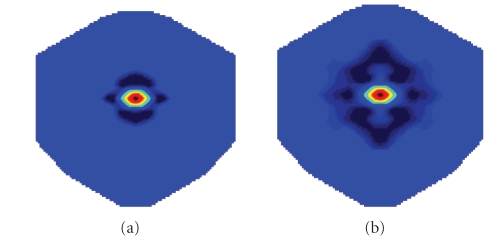
(a) A bell-shaped spatial filter; (b) a learned spatial filter.

In Section 4, a bell-shaped spatial filter is applied as a preprocessing for the DS features. Equivalently, this prefilter can be viewed as a weighting on the electrodes. Spatially, it resembles a Mexico hat, a positive peak surrounded by a ring of negative peaks (as illustrated in Figure 5(a)).

Our filter learning method further optimizes this prefilter by modifying its shape in the frequency domain (e.g., Figure 3(a)). After the optimization, the spatial influence of the resulting filter remains similar to the prefilter (Figure 5(b)). However, the separation between the positive and the negative peaks of the learned filter increases. This allows signals of lower spatial frequency to pass. Such adaptation helps the filter to extract more discriminative signals from the EEG signals.

An interesting observation is that the spatial filters obtained from the CSP method locally resemble the prefilter we applied for the DS features. As shown in the middle column of Table 1, the filters learned by the CSP method emphasize the electrode directly above the neuronal cluster in charge of the imagination; at the same time, they suppress the contribution from adjacent electrodes.

While our filter learning method employs the prefilter as a prior knowledge and successively refines this knowledge locally, the CSP method arrives at similar results by computing a global filter instead. In the cases where this prior knowledge is accurate, we expect that better filters can be obtained by our method, which eventually leads to lower classification error (e.g., the classification error for subject “av” in Table 2).

### 5.2. Higher-order dynamical system features

In this paper, the covariance is used as a measure of dependence between different regions of the brain. Covariance, however, can only detect second-order dependence between the signals. Other more-powerful measures are needed if one wants to exploit higher-order dynamical system (DS) features of the brain.

Various measures have been explored in the literature. For instance, phase synchronization has also been employed as DS features for classifying BCI signals [9, 10]. Another example is the mutual information [13], but its use in BCI context remains unexplored. In all these cases, however, it is not yet clear how spatial and temporal filters can be learned automatically from the data.

## 6. CONCLUSION

In this paper, we exploited the collective dynamics of the cortex as features for BCI. We also proposed a framework for learning the optimal spatial and temporal filters during the extraction of these features. For 4 of the 5 subjects tested, our automated approach reduces classification errors to less than 10%. This performance is comparable to that of the CSP features obtained with manually tuned parameters. Further comparisons with other filter learning approaches also show the competitive performance of our method. Our results suggest that the dynamical system features combined with filter learning approach are very promising for BCI. More investigation is needed to fully demonstrate its advantage.

## Figures and Tables

**Algorithm 1:**
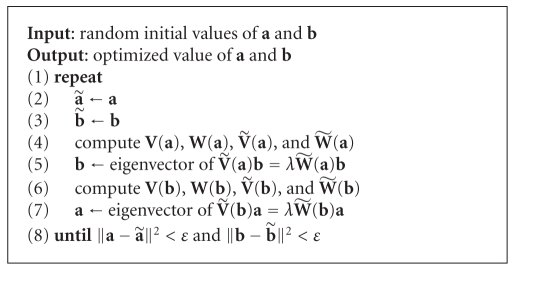
Learning optimal filter **a** and **b**.

**Table 1:** Averaged dynamical system features (DS) and common spatial patterns (CSP) for five subjects (“aa,” “al,” “av,” “aw,” and “ay”) over three-time windows (0–0.5 second, 0.5–2.5 seconds, and 3.5–4.0 seconds) during the motor imagination. The top 20 most discriminative DS features are shown as edges connecting the corresponding electrodes (dots). The most discriminative CSPs for right-hand imagination are plotted as color maps.

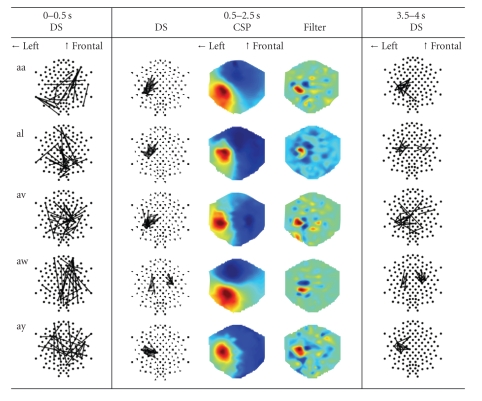

**Table 2:** Classification errors (%) of the CSP, the AR, and the DS features with optimized filters.

**Sb**	**CSP**	**AR**	**DS + S + T**
aa	**8.5** ± **5.4**	**10.5** ± **6.0**	*9.5* ± *5.7*
al	**0.8** ± **1.8**	*1.6* ± *2.5*	2.7 ± 3.1
av	29.1 ± 8.2	*23.3* ± *7.6*	**21.5** ± **7.6**
aw	**3.1** ± **2.8**	7.7 ± 3.8	6.5 ± 4.5
ay	**5.3** ± **3.8**	9.5 ± 4.4	*8.5* ± *5.0*

**Table 3:** Classification errors (%) of the combinations of the CSP, the AR, and the DS feature.

**Sb**	**CSP + AR**	**CSP + DS**	**AR + DS**	**ALL**
aa	*7.6* ± *5.0*	$\begin{array}{c} 7.3\pm 5.1 \end{array}$	7.7 ± 4.7	$\begin{array}{c} 7.3\pm 4.9 \end{array}$
al	1.6 ± 2.3	0.9 ± 1.9	1.6 ± 2.5	*1.5*± *2.2*
av	22.3 ± 7.4	22.5 ± 7.8	$\begin{array}{c} 21.4\pm 7.4 \end{array}$	*21.6* ± *7.1*
aw	3.5 ± 3.2	$\begin{array}{c} 2.8\pm 3.1 \end{array}$	5.2 ± 3.8	*3.4* ± *3.2*
ay	8.9 ± 4.6	**5.5** ± **4.3**	9.1 ± 4.6	*8.7* ± *4.5*

**Table 4:** Classification errors (%) of the DS features before and after applying the learned filters.

**Sb**	**DS**	**DS + S**	**DS + T**	**DS + S + T**
aa	16.7 ± 7.2	14.6 ± 7.0	**9.7** ± **5.7**	9.5 ± 5.7
al	3.7 ± 3.3	**3.2** ± **3.2**	3.6 ± 3.4	2.7 ± 3.1
av	27.3 ± 7.9	25.1 ± 8.0	**21.4** ± **7.9**	21.5 ± 7.6
aw	13.1 ± 6.0	12.1 ± 5.7	**7.5** ± **4.4**	6.2 ± 4.5
ay	11.0 ± 5.3	**9.6** ± **5.0**	9.7 ± 5.1	8.5 ± 5.0

**Table 5:** Classification errors (%) of the CSSP, the CSSSP, and the DS + S + T methods.

**Sb**	**CSSP**	**CSSSP**	**DS + S + T**
aa	14.6 ± 6.2	*11.6* ± *6.3*	**9.5** ± **2.1**
al	*2.3* ± *3.0*	**2.1** ± **2.7**	2.7 ± 3.1
av	32.6 ± 7.6	*31.8* ± *7.7*	**21.5 ± 7.6**
aw	**3.5** ± **3.3**	*6.5* ± *4.3*	*6.5* ± *4.5*
ay	**6.0** ± **3.9**	10.5 ± 5.7	*8.5* ± *5.0*
